# Factors Associated with Postoperative Respiratory Complications following Posterior Spinal Instrumentation in Children with Early‐onset Scoliosis

**DOI:** 10.1111/os.13351

**Published:** 2022-06-10

**Authors:** Ying Zhang, Yingsong Wang, Jingming Xie, Ni Bi, Zhi Zhao, Tao Li, Zhiyue Shi, Tianyi Huang, Bing Gao, Kaiwen Gu, Wuyao Li

**Affiliations:** ^1^ Department of Orthopaedics The 2nd Affiliated Hospital of Kunming Medical University Kunming China

**Keywords:** Complication, Early‐onset scoliosis, Respiratory, Spinal deformity, Surgery

## Abstract

**Objective:**

To investigate the incidence and risk factors of postoperative respiratory complications (PRCs) in children with early‐onset scoliosis (EOS) following posterior spine deformity surgery (PSDS) based on growth‐friendly techniques, so as to help improve the safety of surgery.

**Methods:**

A retrospective study of children with EOS admitted for PSDS based on growth‐friendly techniques from October 2013 to October 2018 was reviewed at a single center. There were 73 children (30 boys, 43 girls) who fulfilled the criteria in this research. The mean age of the patients was 7 ± 6.2 years. Patients were divided into the groups with and without PRCs. Variables that might affect the PRCs during the perioperative period, including general factors, radiographic factors, laboratory factors and surgical factors, were analyzed using univariate analysis to evaluate the potential risk factors. The variables that were significantly different were further analyzed by binary logistic regression analysis to identify the independent factors of PRCs.

**Results:**

All the 73 children included 42 idiopathic scoliosis (57.5%), 12 congenital scoliosis (16.4%), 10 syndromic scoliosis (13.7%) and nine neuromuscular scoliosis (12.3%). PRCs were detected in 16 children (21.9%) with nine different PRCs. The total frequency of detected PRCs was 54, including pleural effusion (25.9%), postoperative pneumonia (20.4%), hypoxemia (18.5%), atelectasis (14.8%), prolonged intubation with mechanical positive pressure ventilatory support (PIMPPVS) (7.4%), bronchospasm (3.7%), reintubation (3.7%), delayed extubation (3.7%) and pneumothorax (1.9%). Results of univariate testing demonstrated that the following six variables were statistically different (*P* < 0.05): nonidiopathic scoliosis, combined with pulmonary comorbidities, pretransferrin < 200 mg/dL, prealbumin < 3.5 g/dL, anesthesia time ≥ 300 min and blood loss to total blood volume ratio (BL/TBV) ≥ 15%. Binary logistic regression analysis confirmed that BL/TBV≥15% (odd ratio OR = 29.188, *P* = 0.010), combined with pulmonary comorbidities (OR = 19.216, *P* = 0.012), pretransferrin < 200 mg/dL (OR = 11.503, *p* = 0.024), and nonidiopathic scoliosis (OR = 7.632, *P* = 0.046) were positively linear correlated with PRCs in children with EOS following PSDS.

**Conclusion:**

PRCs has a higher incidence in children with EOS following PSDS. BL/TBV ≥15%, combined with pulmonary comorbidities, pre‐transferrin < 200 mg/dL, and nonidiopathic scoliosis play an important role for the development of PRCs in this population.

## Graphical abstract

An incidence of 21.9% postoperative respiratory complications (PRCs) was observed in early‐onset scoliosis (EOS) patients after posterior spine deformity surgery (PSDS). The most frequent PRCs was pleural effusion. Risk factors of PRCs were BL/TBV ≥15%, a combination of pulmonary comorbidities, pretransferrin < 200 mg/dL and nonidiopathic scoliosis.

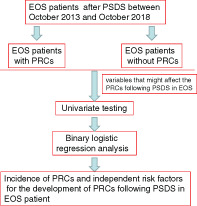

## Introduction

According to both the Scoliosis Research Society and the Pediatric Orthopedic Society of North America, early‐onset scoliosis (EOS) is referred to as curvature of the spine ≥10° in the coronal plane with onset before age 10 years regardless of etiology.^1^ Early diagnosis and appropriate treatment are of utmost importance because spine, thoracic cage and lung development in this age group occur rapidly, placing children with EOS at significant risk for rapid deformity progression and thoracic constraints, ultimately leading to impaired pulmonary function. In addition, children with EOS may also be associated with cardiopulmonary and/or gastrointestinal pathological anomalies, which can exacerbate pulmonary insufficiency.^1^ Thus, compared with adolescent idiopathic scoliosis, children with EOS demonstrate higher mortality and lower quality of life.

Previous studies have shown that bracing and casting may slow the progression of spinal deformity, but up to a one‐third of conservatively managed children with EOS eventually undergo surgery.^2^ Therefore, children with severe progressive EOS require early surgical intervention to prolong their life and maintain a better quality of life. When treating children with EOS who do not respond to brace or cast treatments, the surgery based on growth‐friendly techniques is preferable to fusion surgery because it has less interference with the normal development of the children's spine, thoracic cage and lung.

Although remarkable advances have been made in growth‐friendly techniques and implants over the past decades, most of which are posterior procedures due to their fewer adverse effects on postoperative respiratory function, the treatment of EOS remains a challenge. Initial reports of these growth‐friendly techniques have demonstrated superior deformity correction and predicted growth maintenance during the treatment period. However, postoperative complication rates remain high (29%–58%). Among these, postoperative respiratory complications (PRCs) are the most common postoperative nonneurological complications following posterior spine deformity surgery (PSDS) for treating EOS.^3^, ^4^, ^5^ PRCs are generally defined as any adverse event that affects the respiratory system during the intraoperative or postoperative period. PRCs can lead to an increased length of hospitalization, worsened patient outcomes, and higher hospital and postoperative costs.^3^, ^4^, ^5^ Therefore, it is important to determine the various factors that put children with EOS at increased risk of PRCs after PSDS. This will allow for an optimized perioperative management strategy, improved allocation of clinical resources, and better patient experience. However, there is a paucity of studies correlating PRCs with the characteristics of children diagnosed with EOS. In addition, there is no standardized definition of PRCs in the existing literature, which makes the clinical research results lack any clinical guidance significance.

The current study analyzed the data of children with EOS undergoing PSDS at a single institution in the past 5 years with a minimum of 2 years of follow‐up with the objective of: (i) determining the incidence of PRCs; (ii) cataloging the kinds and frequency of PRCs; and (iii) identifying the risk factors for PRCs. We believe that our data could help spine surgeons to guide perioperative management and surgical planning, thus reducing the probability of PRCs after PSDS.

## Materials and Method

### Patient Cohort

Institutional Review Board approval (No. PJ‐2021‐37) was obtained by the Scientific and Research Ethics Committee of the Second Affiliated Hospital of Kunming Medical University before the start of this retrospective study. Written consent was obtained from the children's guardians. Inclusion criteria: (i) children with EOS; (ii) the deformity continues to progress and the coronal Cobb angle of the main curve ≥40° after regular conservative treatment or deformities that are predicted to have a high risk for progression; (iii) underwent PSDS based on growth‐friendly techniques (including various posterior approach growth‐friendly techniques); (iv) children with complete preoperative and postoperative imaging and clinical data; and (v) a minimum of 2 year of follow‐up after the initial PSDS. Exclusion criteria: final arthrodesis surgery.

The databases at our center, including general factors, radiographic factors, laboratory factors and surgical factors, were reviewed, and 10 variables were selected to compare the differences between the groups with and without PRCs in children with EOS who had undergone PSDS between October 2013 and October 2018 at our center. The presence or absence of PRCs was determined by two respiratory physicians using an independent double‐blind method.

### Surgical Techniques

All PSDSs were generally categorized into two types according to the type of correction technique applied: distraction‐based and other techniques (including compression‐based, growth‐guidance and hybrid techniques).

All surgeries were performed under general anesthesia in the prone position. Neurophysiological monitoring of somatosensory evoked potentials and MEPs were performed routinely. Two to three small incisions for a minimally invasive posterior approach to the spine were utilized in the determined spine levels for the upper, lower instrumented vertebra and apex vertebrae. Pedicle screw insertion with the freehand technique for proximal, apex and distal anchors were performed according to the preoperative plan. The rods, which were contoured for maximal curve correction as well as the establishment of appropriate lumbar lordosis and thoracic kyphosis, were placed submuscularly from proximal to distal. Then, proper correction maneuvers were applied to the spinal curvature to restore the trunk balance. When distraction‐based techniques were performed, the lengthening interval mostly ranged from 6 to 12 months before the final arthrodesis surgery.

### General Factors

The general information included gender, etiology and pulmonary comorbidities. We used the C‐EOS classification to describe the etiological characteristics of the children with EOS in this study.^6^ Scoliosis with identified reasons was defined as nonidiopathic scoliosis, and the remaining cases were defined as idiopathic scoliosis. Therefore, the etiology was divided into two broad groups: nonidiopathic and idiopathic scoliosis. The assessment of comorbidity requires a multidisciplinary involvement. All of the children were divided into two broad groups: combined with pulmonary comorbidities or not.

### Radiographic Factors

The radiographic information included coronal major curve angle and sagittal kyphosis angle. We used two variables of C‐EOS classification to describe the radiographic characteristics of the children with EOS.^6^ In the C‐EOS classification, four groups were defined according to the coronal major curve subgrouping: coronal major curves of <20°, 20° to 50°, 51° to 90° and >90°. Accordingly, in this study, the coronal major curve angle was divided into two broad groups: coronal major curve angle greater than 50° or not. Regarding kyphosis, the C‐EOS classification defined a normokyphotic (N) range of 20° to 50°. Kyphosis lower or higher than this extent was defined as hypokyphotic (−) or hyperkyphotic (+). Therefore, kyphosis was also divided into two groups: normokyphotic (N) and abnormokyphotic.

### Laboratory Factors

To analyze the relationship between preoperative nutritional status and PRCs, preoperative transferrin (pretransferrin < 200 mg/dL) and preoperative albumin (prealbumin < 3.5 g/dL) were selected as markers from the preoperative laboratory test results.

### Surgical Factors

Anesthesia time ≥ 300min, types of PSDS and blood loss to total blood volume ratio (BL/TBV≥15%) were selected as surgical factors that might affect the PRCs following PSDS. Anesthesia time was defined as the time elapsed from intubation under general anesthesia until the patient was removed from the operating room following surgery. All surgeries were posterior‐only approach with various growth‐friendly techniques, including distraction‐based techniques, compression‐based techniques, growth‐guidance techniques and hybrid techniques. Among them, the distraction‐based technique predominated. Therefore, the types of PSDS were divided into two groups: the distraction‐based technique group and the other techniques group. Additionally, in view of the different physiological characteristics between children and adults, BL/TBV was selected to evaluate the intraoperative blood loss.

### Observation Index

#### *PRCs*

PRCs are generally considered be comprised of any adverse event that causes a clinically relevant and identifiable pulmonary alteration that affects the respiratory system in either the intraoperative or the postoperative period, including postoperative pneumonia, pleural effusion, hypoxemia, atelectasis, bronchospasm, pneumothorax, reintubation, delayed extubation and prolonged intubation with mechanical positive pressure ventilatory support (PIMPPVS). We used standardized definitions to determine PRCs following PSDS in children with EOS, which were those described in the statement from the ESA‐ESICM joint taskforce on perioperative outcome measures.^7^ The presence of any perioperative respiratory symptoms or physical findings was recorded. Chest radiographs and/or thoracic ultrasound images were obtained from patients with abnormal cardiopulmonary symptoms and signs to determine the presence of PRCs when necessary:

1. Postoperative pneumonia was considered if two or more serial chest radiographs after surgery with at least one of the following (one radiograph is sufficient for patients with no underlying pulmonary or cardiac disease): (i) new or progressive and persistent infiltrates; (ii) consolidation; (iii) cavitation; and at least one of the following: (a) fever (>38 °C) with no other recognized cause; and (b) abnormal leucocyte count (<4000 or > 12,000/mm^3^); and at least two of the following: (a) new onset of purulent sputum or a change in the characteristic of the sputum, or increased respiratory secretions, or increased suctioning requirements; (b) new onset or worsening cough, or dyspnea, or tachypnea; (c) bronchial breath sounds; or (d) worsening gas exchange (hypoxemia, increased oxygen requirement, increased ventilator demand).
2. Pleural effusion was considered if chest radiograph demonstrated blunting of the costophrenic angle, loss of sharp silhouette of the ipsilateral hemidiaphragm in the upright position, evidence of displacement of the adjacent anatomical structures or (in the supine position) a hazy opacity in one hemithorax with preserved vascular shadows, regardless of the need for treatment.
3. Hypoxemia was considered if arterial blood gas analysis indicated a deficient exchange of oxygen (PO_2_ < 60 mmHg), regardless of the need for treatment.
4. Atelectasis was considered if lung opacification occurred with a shift of the mediastinum, hilum or hemidiaphragm toward the affected area with compensatory overinflation in the adjacent nonatelectatic lung on chest radiograph, regardless of the need for treatment.
5. *Bronchospasm*: Bronchospasm was considered if any episode of wheezing was associated with acute respiratory symptoms and was relieved by bronchodilators.
6. Pneumothorax was considered if air was present in the pleural space with no vascular bed surrounding the visceral pleura on chest radiograph, regardless of the need for treatment.
7. Delayed extubation, reintubation or prolonged intubation with mechanical positive pressure ventilatory support (PIMPPVS) was defined as a continuation of postoperative ventilation beyond 24 hours in line with the previous literature. Reintubation was defined as a need for reintubation within 24 hours of extubation. Prolonged intubation with mechanical positive pressure ventilatory support (PIMPPVS) was defined as a continuation of postoperative ventilation beyond 48 hours.

### Statistical Analysis

Ten variables that might affect the PRCs following PSDS in children with EOS were analyzed using SPSS software version 19.0 (Chicago, IL, USA). In the univariate testing, categorical variables were analyzed using Pearson chi‐square tests and Fisher exact tests where appropriate to examine potential risk factors. Factors with a *P* value < 0.05 were considered statistically significant and included as potential risk factors in binary logistic regression analysis to identify significant independent risk factors for PRCs following PSDS in children with EOS. Statistical significance was accepted when the *P* values were less than 0.05 in binary logistic regression analysis. We generated a receiver operating characteristic (ROC) curve using predicted probability values from the logistic regression model. When the area under the curve (AUC) is greater than 50%, the predicted probability value is considered to be more accurate than chance.

## Results

### Demographics

A retrospective consecutive series of 73 children with EOS who were treated by PSDS were included in this study. Of the 73 children, 30 (30/73, 41.1%) were boys and 43 (43/73, 58.9%) were girls with a mean age of 7 ± 6.2 years (range, 6 years and 2 months–11 years and 4 months) at initial PSDS. Mean follow‐up was 22.5 months (range, 12–49 months). The mean BMI was 24.05 ± 2.52 kg/m^2^. There were 42 idiopathic scoliosis (42/73, 57.5%), 12 congenital scoliosis (12/73, 16.4%), 10 syndromic scoliosis (10/73, 13.7%), and nine neuromuscular scoliosis (9/73, 12.3%). Of the 73 children, 58 (58/73, 79.5%) underwent initial PSDS and 15 (15/73, 20.5%) underwent rod‐lengthening procedures. Twenty‐eight (28/73, 38.4%) and 45 (45/73, 61.6%) children underwent distraction‐based and other techniques, respectively, according to the type of PSDS. The initial and last follow‐up coronal and sagittal main curve magnitudes were 72.16° ± 20.21° and 30.28° ± 18.67°; 54.26° ± 13.33° and 44.58° ± 16.53°, respectively (Table 1).

**TABLE 1:** General information and clinical data

Parameter	Data
Age at initial PSDS (years)	7 ± 6.2
Gender, n (%)	
Boys	30 (41.1)
Girls	43 (58.9)
BMI (kg/m^2^)	24.05 ± 2.52
Etiology, n (%)	
Idiopathic scoliosis	42 (57.5)
Congenital scoliosis	12 (16.4)
Syndromic scoliosis	10 (13.7)
Neuromuscular scoliosis	9 (12.3)
Initial surgery (initial/none)	58/15
Coronal main curve magnitude (°)	
Initial	72.16 ± 20.21
Last follow‐up	30.28 ± 18.67
Sagittal maximum angle of kyphosis (°)	
Initial	54.26 ± 13.33
Last follow‐up	44.58 ± 16.53
Type of PSDS (distraction‐based/other techniques)	28/45

Abbreviations: BMI, body mass index; PSDS, posterior spine deformity surgery based on growth‐friendly techniques.

### Incidence of Postoperative Respiratory Complications

PRCs were detected in 16 children (16/73, 21.9%; 6 boys and 10 girls) with nine different PRCs. Some children developed more than one complication. The total frequency of detected PRCs was 54 in this study. The most frequent PRC was pleural effusion (14/54, 25.9%). Other pathologies were postoperative pneumonia (11/54, 20.4%), hypoxemia (10/54, 18.5%), atelectasis (8/54, 14.8%), PIMPPVS (4/54, 7.4%), bronchospasm(2/54, 3.7%), reintubation (2/54, 3.7%), delayed extubation (2/54, 3.7%) and pneumothorax (1/54, 1.9%) (Table 2).

**TABLE 2:** Frequency and percentages of different PRCs among the 16 patients who developed PRCs

PRCs	Frequency	Percentage (%)
Pleural effusion	14	25.9
Postoperative pneumonia	11	20.4
Hypoxemia	10	18.5
Atelectasis	8	14.8
PIMPPVS	4	7.4
Bronchospasm	2	3.7
Reintubation	2	3.7
Delayed extubation	2	3.7
Pneumothorax	1	1.9
Total	54	100

Abbreviations: PRCs, postoperative respiratory complications; PIMPPVS, prolonged intubation (>48 h) with mechanical positive pressure ventilatory support.

### Univariate Testing between the Groups of Children with and without Postoperative Respiratory Complications

In general factors, compared with children without PRCs, children with PRCs were strongly positively associated with nonidiopathic scoliosis (*P* = 0.003). These patients were also more likely to have pulmonary comorbidities (*P* = 0.003). Regarding laboratory factors, the blood biochemical indicators, pretransferrin (*P* = 0.002) and prealbumin (*P* = 0.016), which reflects nutritional status and oxygen carrying status, were lower in children with PRCs. Regarding the surgical factors, more anesthesia time (*P* = 0.007) was consumed, and more BL/TBV (*P* = 0.000) was detected in children with PRCs than in those without PRCs. No significant difference in gender, major curve angle, kyphosis, or types of PSDS was observed (Table 3).

**TABLE 3:** Catalogue of cases with or without PRCs on variables

Group N = 73	No. of patients	No. of patients with PRCs	No. of patients without PRCs	*X* ^2^	*p* values
Gender	Male (N = 30)	6	24	0.060	0.807
	Female (N = 43)	10	33		
Etiology	Non‐idiopathic (N = 31)	12	19	8.877	0.003*
	Idiopathic (N = 42)	4	38		
Combined with pulmonary comorbidities	Yes (N = 21)	10	11	9.121	0.003*
	No (N = 52)	6	46		
Major curve angle (>50°)	Yes (N = 44)	9	35	0.139	0.710
	No (N = 29)	7	22		
Kyphosis (N)	Yes (N = 20)	4	16	0.059	0.808
	No (N = 53)	12	41		
Pretransferrin (<200 mg/dL)	Yes (N = 34)	13	21	9.901	0.002*
	No (N = 39)	3	36		
X7: Prealbumin (<3.5 g/dL)	Yes (N = 23)	9	14	5.813	0.016*
	No (N = 50)	7	43		
X8: Anesthesia time (≥300 min)	Yes (N = 25)	11	14	7.264	0.007*
	No (N = 48)	5	43		
X9: Types of PSDS	Distraction‐based (N = 28)	8	20	1.175	0.278
	Other (N = 45)	8	37		
X10: BL/TBV (≥15%)	Yes (N = 31)	13	18	12.616	0.000*
	No (N = 42)	3	39		

Abbreviations: BL/TBV, blood loss to total blood volum ratio.

* *p* < 0.05, statistically significant difference between the two groups.

### Binary Logistic Regression between the Variables with Significant Differences in Univariate Testing

**Fig. 1:**
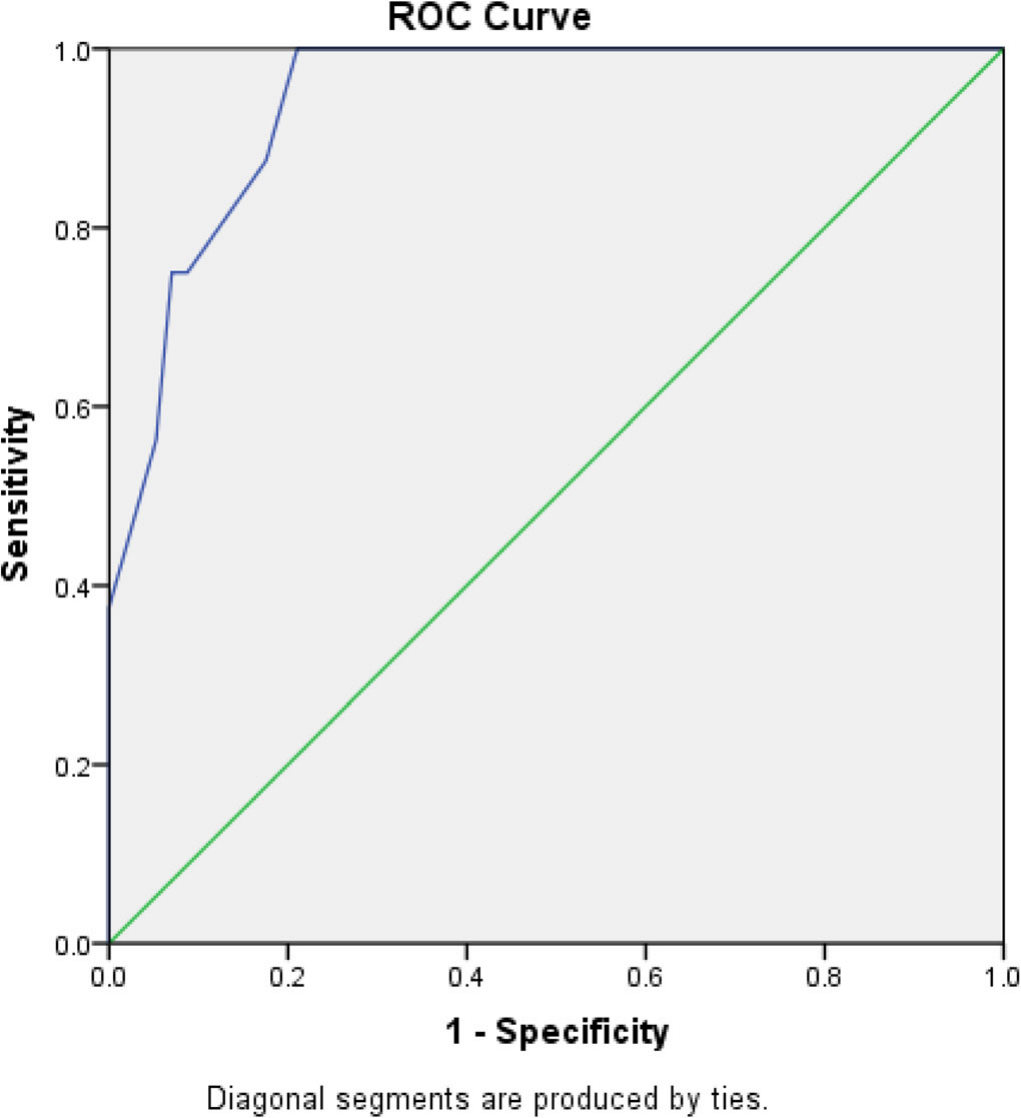
The receiver operating characteristic (ROC) curve based on predicted probability of the logistic regression model has been generated. The horizontal axis and the vertical axis represent specificity and sensitivity, respectively. The blue line is the ROC curve of the predicted probability value (*P* < 0.05). The area under the curve (AUC) is 94.3%, which is greater than 50%, and the curve is close to the upper left corner. This means that the predicted probability value in this research is more accurate than chance

The six variables that were significantly different were further analyzed using binary logistic regression analysis. The results of binary logistic regression indicated that children with EOS following PSDS were positively linear correlated with the following factors with regard to the PRCs: BL/TBV ≥15% (B = 3.374, odds ratio OR = 29.188, *P* = 0.010), combined with pulmonary comorbidities (B = 2.956, OR = 19.216, *P* = 0.012), pretransferrin < 200 mg/dL(X6)(B = 2.443, OR = 11.503, *P* = 0.024), and nonidiopathic scoliosis (B = 2.032, OR = 7.632, *P* = 0.046). Compared with standard partial regression coefficients (OR), BL/TBV ≥15% was the most significant factor affecting PRCs following PSDS in children with EOS (Tables 4 and 5). The ROC curve based on the predicted probability of the logistic regression is shown in Fig. 1, and the area under the curve was 0.943 (95% CI, 0.893 to 0.993) (Fig. 1).

**TABLE 4:** Weight assignment of variables

Factors	Value assignment
Etiology	1 = “Non‐idiopathic”, 2 = “Idiopathic”
Combined with pulmonary comorbidities	1 = “Yes”, 2 = “No”
Pretransferrin (<200 mg/dL)	1 = “Yes”, 2 = “No”
Prealbumin (<3.5 g/dL)	1 = “Yes”, 2 = “No”
Anesthesia time (≥300 min)	1 = “Yes”, 2 = “No”
BL/TBV (≥15%)	1 = “Yes”, 2 = “No”

**TABLE 5:** Results using binary logistic regression analysis method

Variables	B	S.E.	Wald	OR	Sig.	95% CI for EXP (B)	
						Lower	Upper
Etiology	2.032	1.020	3.970	7.632	0.046*	1.034	56.342
Combined with pulmonary comorbidities	2.956	1.178	6.291	19.216	0.012*	1.908	193.524
Pretransferrin (<200 mg/dL)	2.443	1.083	5.085	11.503	0.024*	1.377	96.121
BL/TBV (≥15%)	3.374	1.310	6.636	29.188	0.010*	2.241	380.183
Coefficient	−7.915	1.973	16.095	0.000			

Abbreviations: OR odds ratio; SE standard error.

* *P* < 0.05, statistically significant.

## Discussion

The main result of this study indicated that the incidence of PRCs was 21.9%. Univariate analysis demonstrated that the general, radiographic, laboratory and surgical factors significantly associated with PRCs were nonidiopathic scoliosis, combined with pulmonary comorbidities, pretransferrin <200 mg/dL, prealbumin <3.5 g/dL, anesthesia time > 300 min and blood loss to total blood volume ratio ≥ 15%. In multivariate analysis, statistically significant independent predictors of PRCs were blood loss to total blood volume ratio ≥ 15%, combined with pulmonary comorbidities, pretransferrin <200 mg/dL and nonidiopathic scoliosis.

### The incidence of postoperative respiratory complications

The previous literature indicated different incidences of PRCs and used different definitions of PRCs, which makes the results lack clinical guidance significance. To obtain more reliable results, we used standardized definitions of PRCs in this study.^7^ We examined 73 children with EOS following PSDS, and we found a higher incidence of PRCs (21.9%) than other investigators, who demonstrated an average incidence of approximately 11%–15% for PRCs associated with congenital or nondegenerative scoliosis^8^, ^9^

The high incidence of PRCs in children with EOS is likely to be caused by anatomical changes. In contrast to other types of scoliosis, EOS is a progressive spinal deformity that occurs during the critical period of lung development (within the first 10 years) and is generally associated with the development of restrictive thoracic cages, ribs, and sternums, limiting the number of alveoli and the lung volume. This eventually leads to lung defects, as demonstrated by a decrease in lung volume, vital capacity, and chest wall compliance based on pulmonary function testing. In addition, this progressive spinal deformity is likely to affect the position of various organs and the maximal strength of the respiratory muscle groups residing in the chest cavity, causing mechanical airway obstruction. In most children with EOS, restrictive lung disease occurs due to a reduced intrathoracic volume and increased chest wall stiffness over time. The obstructive mechanism is the compression of a main stem bronchus from the “intrathoracic spinal hump” and mediastinal contents.

Children with EOS may also develop symptoms of lower airway obstruction, which may be the result of chronic airway inflammation and reduced bronchial cilia function secondary to the poor clearance of secretions. The changes in anatomy due to spinal deformity ultimately led to changes in exercise capacity and breathing patterns. Furthermore, patients usually experience severe pain after surgery. These patients are likely to have shallower breathing, thus increasing the possibility of progressive atelectasis. In addition, narcotic agents and analgesics have a similar effect.^10^, ^11^, ^12^, ^13^ All of these pathological changes mentioned above contribute to a higher incidence of PRCs in children with EOS following PSDS.

Nine different PRCs were detected, and the total frequency of detected PRCs was 54 in this study. The most frequent PRC was pleural effusion (25.9%). In terms of pathophysiological changes, these complications are mutually causal during their occurrence and development. Massive amounts of fluid input in a relatively short period of time during surgery, surgical manipulation around the pleura and pleural injury during surgery often lead to the development of pleural effusions, pulmonary edema and pneumothorax. Due to surgical trauma, being placed in the prone position may impair the chest wall mechanics and lung pathological changes secondary to spinal deformity, so children with EOS are at increased risk of progressive atelectasis, pneumonia, and hypoxemia. All of these progressive blood oxygen exchange disorders lead to PIMPPVS, delayed extubation and reintubation.^10^, ^11^, ^12^, ^13^ The tendency to become hypoxemic may be related to small lung volumes due to the small thoracic cage size with lung distortion.

### Risk Factors for Postoperative Respiratory Complications following Posterior Spine Deformity Surgery in Children with Early‐onset sSoliosis

#### Blood Loss to Total Blood Volume Ratio (BL/TBV)

In the current study, the regression analysis demonstrated that BL/TBV ≥15% was an independent risk factor for the development of PRCs following PSDS in children with EOS. Compared with adults, children with EOS have different hemodynamic characteristics during surgery. The absolute blood volume is much smaller in low‐weight children with EOS, indicating that even minor absolute blood loss will result in hemodynamic instability. Therefore, this study thinks it is more appropriate to use BL/TBV instead of blood loss volume to evaluate the intraoperative blood loss in this type of research. Intraoperative blood loss is a well‐documented risk factor for mortality and morbidity in EOS surgery. Previous studies have shown that massive blood loss during surgery can cause end‐organ damage and increased postoperative complications, including multiple respiratory complications, in addition to hemodynamic instability.^14^, ^15^

It has long been accepted that blood transfusion can correct the physiologic abnormalities associated with intraoperative blood loss and improve patient outcomes. However, the benefits of blood transfusion may be partially offset by PRCs' adverse effects. The mechanism by which blood transfusion worsens outcomes is unknown. Previous research has proposed some hypotheses about the association between intraoperative blood transfusion and PRCs in patients undergoing surgery. First, low‐weight children with EOS receive massive amounts of fluid in a relatively short period of time, causing tremendous shifts in hydrostatic and osmotic pressures. Second, transfusion results in transfusion‐related immunomodulation because of the infusion of soluble bioactive substances. These bioactive substances activate the immune system, resulting in transfusion‐related lung injury or immune suppression and increasing susceptibility to PRCs. Moreover, the storage of blood products can decrease cellular deformability and increase adhesion to the vascular endothelium, which leads to impaired microvascular flow and reduced oxygen delivery.^14^, ^15^ The results of our study suggested that reducing blood loss during surgery and perioperative blood transfusion help decrease PRCs.

#### Combined with Pulmonary Comorbidities

According to our study, combined with pulmonary comorbidities was an independent risk factor for the development of PRCs following PSDS in children with EOS. It is well established that a high rate of pulmonary comorbidities, which can exacerbate the effect of spinal deformity on pulmonary function, is expected in EOS cohorts.^8^, ^9^ Segreto *et al*. reported that the incidence of pulmonary comorbidities was the highest (55.2%) among the EOS population.^16^ A cluster analysis in this study determined that among the EOS patients with pulmonary disease, 12.2% had pulmonary failure, and 10.5% had restrictive lung disease. According to the results of Segreto *et al*., EOS patients with concurrent neurologic and pulmonary comorbidities were significantly more likely to develop complications (OR = 1.28). This characteristic was more obvious in the nonidiopathic group. In systemic syndromes and/or neuromuscular conditions, the resulting pulmonary comorbidities range from abnormal respiratory responses to hypoxia and hypercarbia, asthma, sleep disordered breathing, aspiration and respiratory functional impairment/failure.^17^ In this group of children, respiratory functional impairment may progress faster among those with progressing scoliosis, which imposes restrictive forces on a respiratory system already impaired by weak respiratory muscles. Moreover, epilepsy and gastroesophageal reflux disease are comorbidities with a higher incidence in other systems except the respiratory system in nonidiopathic EOS. These comorbidities increase the chance of aspiration and increase the incidence of PRCs during the perioperative period. The results of this study suggested that, for each EOS patient, a detailed assessment of each system as well as the sleep and behavioral patterns should be performed, and corresponding treatments should be administered from the initial visit before surgery to decrease PRCs.

#### Transferrin

Our data demonstrated that a lower level of preoperative serum transferrin was an independent risk factor for the development of PRCs following PSDS in children with EOS. The optimization of preoperative nutrition can help reduce perioperative complications, including PRCs, in pediatric scoliosis populations. The serum albumin level and total lymphocyte count have been commonly used as sensitive indicators of nutritional status (malnutrition is commonly defined as a total lymphocyte count <1500–2000 cells/mm^3^, serum albumin level <3.0–3.5 g/dL, or serum transferrin level < 200 mg/dL).^18^ Serum transferrin, prealbumin, and retinol‐binding protein are recognized as rapid turnover proteins because their serum half‐life values are shorter than that of albumin. This characteristic makes them better nutritional biomarkers for the early detection of nutritional deficits. Therefore, transferrin has been identified as a perioperative marker of serum protein and iron status that is useful in the prediction of perioperative morbidity and mortality. Our analysis indicated that it is important to perform a thorough evaluation of nutritional status to decrease PRCs due to perioperative malnutrition.

#### Etiologic Factor

EOS is a group of heterogeneous diseases with highly variable manifestations and is medically complex. To describe and guide optimal care and predict outcomes within the EOS population, Williams *et al*. proposed an EOS classification system.^6^ According to the characteristics of EOS, this classification system contains several key factors, including age, etiology, the severity of the deformity in the coronal and sagittal planes and curve progression. Therefore, the author thinks that it is appropriate to use this classification system to evaluate EOS patients in this research.

Based on the current study, the regression data demonstrated that etiology (nonidiopathic scoliosis) was an independent risk factor for the development of PRCs following PSDS in children with EOS. Neuromuscular scoliosis is the second most prevalent spinal deformity after idiopathic scoliosis. Previous studies have illustrated that PRCs are the most frequent postoperative complications in patients with neuromuscular scoliosis, with a particular risk of aspiration pneumonia. Various potential risk factors for these patients' vulnerability to PRCs have been suggested.^19^ Many individuals with neuromuscular scoliosis have impaired motor function, muscle strength and coordination, especially regarding the respiratory muscles. In addition, concurrent comorbidities in individuals with neuromuscular scoliosis may also increase the risk of PRCs, including seizures, swallowing disorders and gastroesophageal reflux. Similarly, congenital/syndromic scoliosis is related to genetic defects or metabolic disorders and is usually associated with nonskeletal abnormalities, including respiratory system abnormalities. A significant proportion of individuals with congenital/syndromic scoliosis have aspiration and restrictive lung defects. Consequently, some studies have revealed that pulmonary/respiratory complications are major complications that occur following the surgical correction of nonidiopathic scoliosis.^20^ Our result is consistent with those of previous studies and confirms that comprehensive preoperative assessments and therapy of EOS require a multidisciplinary involvement.

### Limitations

The findings of this study should be viewed after considering the following limitations. It is difficult to launch a prospective protocol to reduce the incidence of PRCs, given that only a fraction of EOS children have been treated with PSDS in the low incident EOS population. Also, the findings in this study are also limited by its retrospective nature to draw any strong conclusions. Furthermore, relatively smaller sample size in a single institute may lead to sample bias. Multiple‐center, large‐sample, randomized clinical trials are required to confirm our conclusion in the future. However, the results of this research still offer valuable risk information for surgeons considering intervention with PSDS in children with EOS.

### Conclusion

PRCs are the major postoperative nonneurological complications following PSDS in children with EOS. The incidence of PRCs was 21.9% and the most frequent PRC was pleural effusion. This study showed that BL/TBV ≥15%, combined with pulmonary comorbidities, pre‐transferrin < 200 mg/dL, and nonidiopathic scoliosis were independent risk factors for the development of PRCs following PSDS in EOS population.

Although this research demonstrated some independent risk factors for the development of PRCs, there are still significant unresolved clinical questions need to be found to decrease PRCs. A validated respiratory risk questionnaire would be useful for enabling clinicians to provide evidence‐based care for children with EOS at risk of PRSs following PSDS.

## Author Contributions

Ying Zhang, Yingsong Wang and Jingming Xie contributed to the conception and design of this manuscript, the acquisition of the data, the analysis and the interpretation of the data and the drafting of the manuscript. Ni Bi, Zhi Zhao, Tao Li, and Zhiyue Shi. followed up and collected the data. Wuyao Li, Kaiwen Gu, Bing Gao and Tianyi Huang were responsible for the data collection and radiographic measurements. Tianyi Huang conceived the study and participated in its design and coordination, revised the manuscript critically for important intellectual.

## Funding

Fund from the National Natural Science Foundation of China (NSFC). (Grant No. 82060414; 81860403) and the “Special and Joint Program” of Yunnan Provincial Science and Technology Department & Kunming Medical University (Grant No. 202101AY070001‐150) was received in support of this work. No benefits in any form have been or will be received from a commercial party related directly or indirectly to the subject of this manuscript.
